# Does the operative technique (MIPO vs. ORIF) influence surgical outcomes and complications in proximal humerus fractures? A matched-pair analysis

**DOI:** 10.1007/s00068-026-03212-6

**Published:** 2026-05-11

**Authors:** Christiane Barthel, Franziska Rudolph, Moritz Kraus, Yannik Kalbas, Rodd Russek, Hans-Christoph Pape, Florin Allemann

**Affiliations:** 1 Department of Shoulder and Elbow Surgery, Schulthess Klinik, Lengghalde 2, Zurich, CH-8008 Switzerland; 2 Department of Traumatology, University Hospital Zurich, Zurich, Switzerland; 3 University of Zurich, Zurich, Switzerland; 4 Harald-Tscherne Laboratory of Orthopedic and Trauma Research, University Hospital Zurich, Zurich, Switzerland

**Keywords:** Proximal humerus fracture, MIPO, open reduction and internal fixation, Reduction quality, Operative time, DRoPH Score

## Abstract

**Introduction:**

Proximal humerus fractures account for approximately 4–6% of all fractures and are among the most common fractures in the elderly. Surgical treatment is increasingly applied for displaced and unstable fracture patterns. The two most commonly used techniques are open reduction and internal fixation (ORIF) via a deltopectoral approach and minimally invasive plate osteosynthesis (MIPO) using a deltoid-split or anterolateral approach. ORIF allows direct fracture visualization and anatomical reduction but is associated with more extensive surgical exposure. MIPO aims to preserve soft tissues and vascular supply through indirect reduction techniques. This study compares ORIF and MIPO in proximal humerus fractures with regard to surgical parameters, radiographic outcomes, and complications.

**Methods:**

A retrospective single-center cohort of 392 proximal humerus fractures was analyzed. Seventy-nine patients treated with MIPO were matched to 79 patients treated with ORIF using exact matching for fracture type (Mayo FJD classification) and propensity score matching for age and sex. Primary outcomes included operative time, reduction quality, and complication rates.

**Results:**

The MIPO group showed significantly shorter operative times (97.3 ± 30.8 min vs. 112.2 ± 34.0 min, *p* = 0.004). No significant differences were observed in reduction quality or overall complication rates (MIPO 19.0% vs. ORIF 21.5%). However, functional complications and implant malpositioning showed a non-significant trend toward higher rates in the MIPO group, while hardware-related complications were more frequently observed in the ORIF group.

**Conclusion:**

Our findings support MIPO as a valuable alternative to ORIF for proximal humerus fractures, offering reduced operative time without compromising reduction quality or complication rates. Patient selection, fracture complexity, and surgeon experience should guide technique choice. Appropriate training remains essential, and future prospective studies incorporating functional outcome measures are warranted.

**Level of evidence:**

Level III, retrospective matched-pair-study.

## Introduction

Proximal humerus fractures constitute approximately 4–6% of all fractures and represent one of the most common fractures requiring surgical intervention [1–3]. The management of these fractures has evolved significantly, with surgical fixation increasingly favored for displaced and unstable fracture patterns [1, 2].

Two principal surgical techniques have emerged as standard approaches: traditional open reduction and internal fixation (ORIF) using a deltopectoral approach, with the option to apply a double plate osteosynthesis [4], and minimally invasive plate osteosynthesis (MIPO) utilizing a deltoid-split or modified anterolateral approach [5, 6]. While a deltoid-split approach may also be used for ORIF, the deltopectoral approach remains standard for open plate fixation.

ORIF provides wide exposure and direct fracture visualization, but extensive dissection may potentially affect the soft tissue envelope and, depending on surgical technique, the vascular supply to the humeral head [7, 8]. In contrast, MIPO represents a biological approach emphasizing preservation of the periosteal blood supply and soft tissue envelope through indirect reduction techniques, representing an alternative approach to fracture fixation [9–11]. However, limited visualization inherently restricts reduction and implant positioning options, and the course of the axillary nerve limits safe extension of the approach.

The choice between these techniques remains a subject of ongoing debate [2, 7, 12]. We hypothesized that MIPO would demonstrate comparable reduction quality and complication rates to ORIF while offering advantages in operative time. Therefore, this study aims to compare ORIF and MIPO for proximal humerus fractures, evaluating surgical parameters, radiological outcomes, and complications to inform evidence-based clinical decision-making.

## Materials and methods

In this retrospective analysis, patients aged 16 years or older who underwent treatment for proximal humerus fractures at the University Hospital Zurich between January 2014 and December 2024 were identified from the institutional trauma database. All AO fracture types were eligible for inclusion. Patients were included if they had undergone surgical reduction and internal fixation with a locking plate and had provided written informed consent for the use of clinical and radiological data. Exclusion criteria included missing informed consent, lack of follow-up (e.g., due to death or transfer), and patients primarily managed nonoperatively or by other techniques. The choice of surgical approach was based on surgeon preference; all operating surgeons were experienced in both MIPO and ORIF techniques.

### Surgical technique

MIPO was performed through a transdeltoid lateral approach using a proximal incision over the anterolateral acromion and a distal incision for plate passage. Deep dissection was carried out through the deltoid muscle with protection of the axillary nerve. Reduction was achieved indirectly under fluoroscopic guidance, with cerclage sutures used to secure tuberosity fragments. The Philos locking plate (DePuy Synthes) was inserted submuscularly and screws were placed percutaneously using the dedicated targeting guide. A calcar screw was placed whenever the reduction permitted, particularly in varus fracture patterns.

### Matching process

From this cohort, all patients treated with minimally invasive plate osteosynthesis (MIPO) were identified and matched to a control group treated with open reduction and internal fixation (ORIF). As MIPO was used less frequently, ORIF cases were matched to the MIPO group. Matching was performed using exact matching for fracture type according to the Mayo-FJD classification [13], combined with propensity score matching for age and sex. The Mayo-FJD classification was chosen as it is based on fracture pattern recognition and has demonstrated high intraobserver and interobserver reliability [13].

### Parameters

The study assessed general demographic characteristics, operation time, postoperative reduction quality, and complications. Baseline characteristics included age, sex, fracture type according to the AO/OTA classification [14], the Deltoid Tuberosity Index (DTI) [15], and a score assessing predicted intraoperative reduction difficulty (DRoPH Score; Barthel et al., manuscript under review). Reduction quality was assessed on immediate postoperative radiographs using a three-item composite checklist adapted from Schnetzke et al. [16]: head-shaft displacement (anatomical / ≤5 mm / >5 mm), head-shaft alignment (normal 120–150° / minor varus 110–120° / malreduction < 110° or > 150°), and cranialization of the greater tuberosity (anatomical / ≤5 mm / >5 mm). Each item was scored 0–1, yielding a total score of 0–3, with higher scores indicating better reduction quality.

Complications were categorized into six main groups: (1) Functional complications, defined as movement restrictions and pain, assessed retrospectively based on patient-reported complaints in clinical follow-up notes; (2) Avascular Necrosis (AVN); (3) Hardware-related complications (screw cut-through, screw cut-out, and secondary fragment dislocation (including humeral head displacement)); (4) Non-union; (5) Implant malpositioning (proximal plate positioning, lateral plate offset, or intra-articular screw placement), assessed on immediate postoperative radiographs; and (6) Infection.

### Statistics

Data were analyzed using Python and R V4.5.2. Continuous variables are presented as means ± SD and categorical variables as frequencies. Normality was assessed using the Shapiro–Wilk test. Group comparisons were performed using independent t-tests or Mann–Whitney U tests for continuous variables and Chi-square tests for categorical variables. As matching was performed without replacement at the cohort level, unpaired statistical tests were applied. A p-value < 0.05 was considered statistically significant.

## Results

**Fig. 1:**
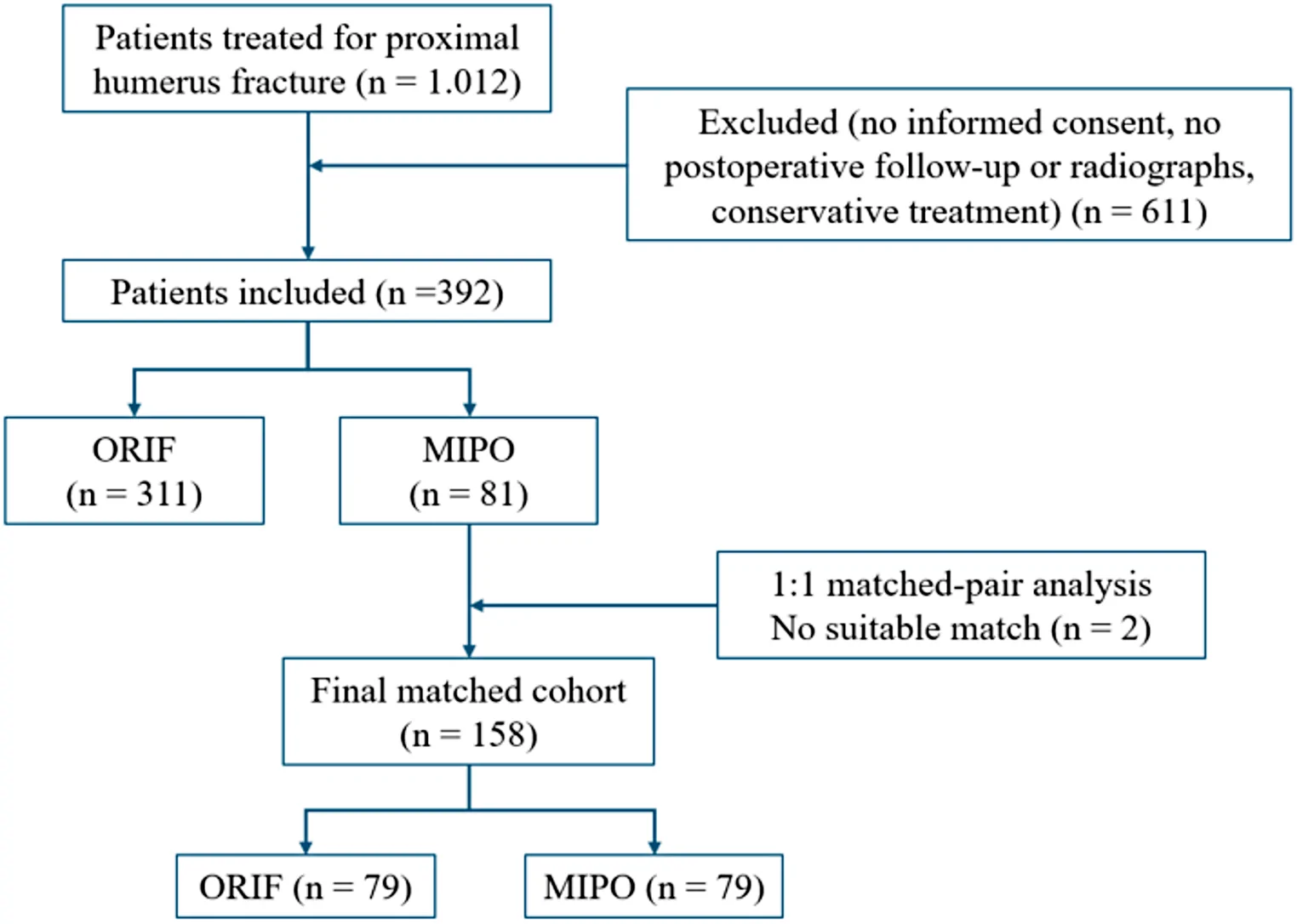
Flowchart of patient selection and matching process

A total of 1,012 patients treated for proximal humerus fractures were identified, of whom 611 were excluded due to missing informed consent, lack of postoperative follow-up or radiographs, or conservative treatment. Of the remaining 392 patients, 311 were treated with ORIF and 81 with MIPO. Following 1:1 matched-pair analysis, one MIPO patient could not be matched and was excluded, resulting in a final cohort of 158 patients with 79 in each group (Fig. 1). Baseline characteristics, including age, sex, AO classification, DTI, and DRoPH score, were similar between the MIPO and ORIF groups. The median follow-up was approximately 12 months in both groups. Age (MIPO: 65.4 ± 18.8, ORIF: 63.8 ± 17.6, range 17–98 years), sex (MIPO: 39.2% male, 60.8% female; ORIF: 35.4% male, 64.6% female), fracture type distribution (AO classification A 34.2%, B 15.2% and C 50.6%), DTI (MIPO: 1.51 ± 0.20, ORIF: 1.47 ± 0.17), and DRoPH score (MIPO: 2.30, median 2; ORIF: 2.77, median 2) showed no significant differences.

Operative time was significantly shorter in the MIPO group (97.3 ± 30.8 min) compared to the ORIF group (112.2 ± 34.0 min, *p* = 0.004), with a median of 99.0 vs. 104.0 min (*p* = 0.013). No significant differences were found in the reduction score (MIPO: 2.61 ± 0.63, ORIF: 2.58 ± 0.73) and complication rates (MIPO: 19.0%, ORIF: 21.5%) (*p* = 0.815). (Table 1)

**Table 1:** Comparison of MIPO vs. ORIF for proximal humerus fractures: baseline characteristics, operative time, and postoperative outcomes

	MIPO	ORIF	*p*-value
*n*	79	79	
Age (mean ± SD)	65.37 ± 18.83	63.80 ± 17.59	0.589
Male n / %	31 / 39.2%	28 / 35.4%	
Female n / %	48 / 60.8%	51 / 64.6%	
AO-classification A	27 / 34.2%	27 / 34.2%	1.000
AO-classification B	12 / 15.2%	12 / 15.2%	1.000
AO-classification C	40 / 50.6%	40 / 50.6%	1.000
DTI (mean ± SD)	1.51 ± 0.20	1.47 ± 0.17	0.238
DRoPH-Score (mean / median)	2.30 / 2	2.77 / 2	0.160
Operative Time (mean ± SD)	97.25 ± 30.83	112.20 ± 34.00	0.004
Operative Time (median in min)	99	104	0.013
Reduction Score (mean ± SD)	2.61 ± 0.63	2.58 ± 0.73	0.815
Complications	15 / 19.0%	17 / 21.5%	0.843

Of the 158 included patients, 27 had AO type A fractures, 12 had AO type B fractures, and 40 had AO type C fractures per group, for a total of 54 type A, 24 type B, and 80 type C fractures across both groups. Baseline characteristics (age, sex, DTI, DRoPH score) were similar between the MIPO and ORIF groups for all fracture types (Tables 2, 3 and 4). The only significant difference between the groups was in operative time. For B-fractures, the MIPO group showed a shorter median operative time (87.5 min vs. 127.5 min, *p* = 0.069), which did not reach statistical significance. For C-fractures, the MIPO group had a significantly shorter mean operative time (99.50 ± 27.80 min vs. 115.10 ± 35.94 min, *p* = 0.033), while the difference in median operative time did not reach significance (104.0 vs. 105.0 min, *p* = 0.104). No significant differences were observed in reduction scores or complication rates in any fracture type.

**Table 2:** Comparison of MIPO vs. ORIF in proximal humerus fractures: baseline characteristics, operative time, and postoperative outcomes by AO fracture type A

A-Fractures:			
	MIPO	ORIF	*p*-value
n	27	27	
Age (mean ± SD)	60.33 ± 19.96	57.63 ± 17.19	0.596
Male n / %	14 / 51.9%	13 / 48.1%	
Female n / %	13 / 48.1%	14 / 51.9%	
AO-classification A	27 / 100.0%	27 / 100.0%	1.000
AO-classification B	0 / 0.0%	0 / 0.0%	1.000
AO-classification C	0 / 0.0%	0 / 0.0%	1.000
DTI (mean ± SD)	1.53 ± 0.16	1.51 ± 0.19	0.652
DRoPH-Score (mean / median)	0.44 / 0	0.81 / 0	0.199
Operative Time (mean ± SD)	95.30 ± 28.43	104.78 ± 29.07	0.231
Operative Time (median in min)	98.0	98.0	0.368
Reduction Score (mean ± SD)	2.89 ± 0.32	2.70 ± 0.54	0.132
Complications	4 / 14.8%	6 / 22.2%	0.728

**Table 3:** Comparison of MIPO vs. ORIF in proximal humerus fractures: baseline characteristics, operative time, and postoperative outcomes by AO fracture type B

B-Fractures:			
	MIPO	ORIF	*p*-value
n	12	12	
Age (mean ± SD)	61.75 ± 20.11	59.75 ± 17.79	0.799
Male n / %	6 / 50.0%	4 / 33.3%	
Female n / %	6 / 50.0%	8 / 66.7%	
AO-classification A	0 / 0.0%	0 / 0.0%	1.000
AO-classification B	12 / 100.0%	12 / 100.0%	1.000
AO-classification C	0 / 0.0%	0 / 0.0%	1.000
DTI (mean ± SD)	1.46 ± 0.13	1.47 ± 0.19	0.935
DRoPH-Score (mean / median)	1.75 / 2	2.08 / 2	0.596
Operative Time (mean ± SD)	94.17 ± 45.32	119.25 ± 37.38	0.153
Operative Time (median in min)	87.5	127.5	0.069
Reduction Score (mean ± SD)	2.67 ± 0.49	2.75 ± 0.62	0.719
Complications	3 / 25.0%	3 / 25.0%	1.000

**Table 4:** Comparison of MIPO vs. ORIF in proximal humerus fractures: baseline characteristics, operative time, and postoperative outcomes by AO fracture type C

C-Fractures:			
	MIPO	ORIF	*p*-value
n	40	40	
Age (mean ± SD)	69.85 ± 16.96	69.17 ± 16.47	0.857
Male n / %	11 / 27.5%	11 / 27.5%	
Female n / %	29 / 72.5%	29 / 72.5%	
AO-classification A	0 / 0.0%	0 / 0.0%	1.000
AO-classification B	0 / 0.0%	0 / 0.0%	1.000
AO-classification C	40 / 100.0%	40 / 100.0%	1.000
DTI (mean ± SD)	1.50 ± 0.24	1.44 ± 0.15	0.212
DRoPH-Score (mean / median)	3.73 / 4	4.30 / 4	0.083
Operative Time (mean ± SD)	99.50 ± 27.80	115.10 ± 35.94	0.033
Operative Time (median in min)	104.0	105.0	0.104
Reduction Score (mean ± SD)	2.40 ± 0.74	2.45 ± 0.85	0.780
Complications	8 / 20.0%	8 / 20.0%	1.000

There were no significant differences in complication rates between the MIPO and ORIF groups. However, some trends were observed. The incidence of functional complications (movement restriction and pain) was higher in the MIPO group (7 vs. 3, *p* = 0.328). Hardware-related complications occurred more frequently in the ORIF group (5 vs. 11, *p* = 0.186). Implant malpositioning was more common in the MIPO group (2 vs. 0, *p* = 0.497). All other complications, including head necrosis, non-union, and infection, showed no significant differences between the groups (*p* = 1.000). Hardware-related complications occurred predominantly in type C fractures in both groups. In the ORIF group, these comprised screw cut-through (*n* = 2) and secondary fragment dislocation (*n* = 9), with a notable trend toward more hardware failures in type A fractures (5 vs. 0, *p* = 0.051). In the MIPO group, hardware complications included screw cut-through (*n* = 3), screw cut-out (*n* = 1), and secondary fragment dislocation (*n* = 1), all occurring in type C fractures. (Table 5)

**Table 5:** Comparison of complications between MIPO and ORIF

	MIPO (*n* = 79)	ORIF (*n* = 79)	*p*-value
Functional	7	3	0.328
Head Necrosis	1	2	1.000
Hardware related	5	11	0.186
Non-union	0	1	1.000
Implant malpositioning	2	0	0.497
Infection	1	0	1.000
Total	15 / 19.0%	17 / 21.5%	0.843

## Discussion

This retrospective matched-cohort study compared MIPO with ORIF for proximal humerus fractures, demonstrating that MIPO offers significant operative time advantages while maintaining comparable reduction quality and complication rates across different fracture patterns.

### Operative time

The MIPO group showed significantly shorter operative time. This advantage was most pronounced in complex fractures, particularly in type C fractures. These findings contrast with reports suggesting MIPO requires longer operative times due to indirect reduction demands [9, 12]. This efficiency stems from avoiding extensive deltopectoral dissection [10, 17], streamlined submuscular plate insertion once mastered [18] and our institution’s 10-year accumulated MIPO experience. The shorter operative time benefits elderly patients with comorbidities by reducing anesthesia duration and physiological stress [1, 2], while potentially decreasing infection risk and healthcare costs [19].

### Radiological outcomes and quality of reduction

Despite indirect reduction techniques, MIPO achieved comparable reduction quality scores to ORIF across all AO classifications, including complex type C fractures [6, 10]. Modern fluoroscopic imaging enables precise assessment [17], while percutaneous reduction tools effectively restore anatomy without compromising quality [9, 12]. The preservation of fracture hematoma and soft tissue attachments may further stabilize fragments during healing [18].

### Complications

Overall complication rates were comparable, consistent with recent meta-analyses [20, 21]. However, notable trends emerged:

Functional complications were more frequently observed in the MIPO group and may be related to suboptimal fragment positioning not apparent on fluoroscopy [12], the deltoid-split approach with potential muscle irritation, or increased soft tissue swelling at the fracture site due to preservation of the periosteal hematoma [18].

Hardware-Related Complications: ORIF demonstrated more hardware failures (11 vs. 5), with a particularly pronounced trend in type A fractures (5 vs. 0, *p* = 0.051), potentially due to compromised reduction stability and vascular disruption from periosteal stripping [2, 8]. MIPO’s soft tissue preservation may provide superior biological healing conditions [9, 12].

Implant Malpositioning: Exclusively observed in MIPO, this highlights the technical demands of limited visualization [12] and underscores the importance of adequate training and high-quality fluoroscopic imaging [17, 18].

Avascular Necrosis, Non-Union, and Infection: The comparable rates of AVN, non-union, and infection between groups are particularly significant. While open approaches theoretically carry higher AVN risk due to vascular disruption [8], our equivalent rates suggest that fracture pattern and patient factors (age, bone quality, displacement) may play more dominant roles than surgical approach [2, 22]. The similar non-union and infection rates indicate that MIPO’s soft tissue preservation does not translate to measurably superior healing or infection prevention in our cohort [19, 21].

### Fracture pattern

Subgroup analysis revealed MIPO’s operative time advantage was most pronounced in type C fractures, with no significant differences in type A or type B fractures. This suggests MIPO’s efficiency benefits are greatest in complex patterns requiring extensive open dissection [6, 10], while the time saved in simple two-part fractures may be offset by indirect reduction demands.

Although reduction quality and complication rates were comparable across all fracture types, individual fracture morphology may still influence the suitability of MIPO. In particular, severe comminution increases reduction difficulty, which in the setting of limited visualization may result in suboptimal implant positioning. In such cases, an open approach may facilitate more reliable implant placement and tuberosity restoration. Accordingly, careful case selection remains essential, especially during the early phase of the MIPO learning curve [12].

## Limitations

This study has both strengths and limitations. The strengths include the use of propensity score matching to control for confounding variables, the relatively large matched cohort from a high-volume trauma center, and the comprehensive 10-year study period capturing institutional experience evolution. The detailed complication categorization and objective reduction quality assessment provide nuanced insights beyond simple dichotomous outcomes. Furthermore, the DRoPH score used to assess reduction difficulty has not yet been externally validated, which may limit its generalizability.

Among the limitations, the retrospective design cannot rule out a certain selection bias, as surgeon preference, patient characteristics, and the progressive adoption of MIPO over the study period may have influenced technique selection despite our matching efforts. Also, the lack of functional outcome scores (e.g., Constant-Murley score, DASH score) limits our ability to assess patient-reported outcomes and shoulder function, which represent critical endpoints in fracture management. Follow-up duration variability and incomplete long-term data may have underestimated late complications such as AVN, which can manifest months to years after surgery. Additionally, the study was conducted at a single academic center with substantial experience in MIPO; therefore, institutional factors and the level of surgeon experience may have influenced the observed outcomes, potentially limiting generalizability to centers with different workflows or lower familiarity with minimally invasive techniques.

The relatively small sample sizes within fracture subgroups (particularly type B fractures, *n* = 12) may have limited statistical power to detect significant differences in complications. Future multicenter prospective studies with standardized functional outcome assessment and longer follow-up would provide more robust evidence for clinical decision-making.

## Conclusions

Our findings support MIPO as a valuable alternative to ORIF for proximal humerus fractures, demonstrating reduced operative time without compromising reduction quality or complication rates. This operative time advantage is particularly pronounced in complex fracture patterns and provides meaningful benefits for patients and surgical teams alike. However, the observed trends toward higher functional complications and implant malpositioning in the MIPO group highlight the importance of appropriate patient selection and adequate surgical training. The choice between MIPO and ORIF should be individualized, taking into account fracture complexity, bone quality, and surgeon experience. In cases with poor bone quality, MIPO may be advantageous as indirect reduction minimizes direct manipulation of fragile fragments, reducing the risk of iatrogenic comminution. Conversely, cases with severe comminution or destruction of the greater tuberosity may benefit from the direct visualization offered by an open approach. Future prospective studies incorporating standardized functional outcome measures are warranted to further define the role of each technique.

## Data Availability

No datasets were generated or analysed during the current study.
